# Ultrastructural Mucosal Response to Dupilumab in Chronic Rhinosinusitis With Nasal Polyps: A Pilot Study

**DOI:** 10.1002/clt2.70173

**Published:** 2026-04-27

**Authors:** Francesco Giombi, Gian Marco Pace, Elena Vezzoli, Fabio Grizzi, Fabio Pasqualini, Michele Cerasuolo, Enrico Heffler, Giovanni Paoletti, Francesca Puggioni, Giuseppe Mercante, Giuseppe Spriano, Giorgio Walter Canonica, Luca Malvezzi

**Affiliations:** ^1^ Otorhinolaryngology Head & Neck Surgery Unit Casa di Cura Humanitas San Pio X Milan Italy; ^2^ Otorhinolaryngology Unit IRCCS Humanitas Research Hospital Milan Italy; ^3^ Advanced Light and Electron Microscopy BioImaging Centre (ALEMBIC) IRCCS San Raffaele Scientific Institute Milan Italy; ^4^ Department of Biomedical Sciences Humanitas University Milan Italy; ^5^ Department of Immunology and Inflammation IRCCS Humanitas Research Hospital Milan Italy; ^6^ Personalized Medicine, Asthma and Allergy IRCCS Humanitas Research Hospital Milan Italy; ^7^ Department of Otolaryngology‐­Head and Neck Surgery IRCCS Azienda Ospedaliero‐Universitaria Di Bologna Bologna Italy; ^8^ Alma Mater Studiorum‐Università di Bologna Bologna Italy

**Keywords:** chronic rhinosinusitis, dupilumab, electron microscopy, nasal polyps, rhinology

## Abstract

**Background:**

As monoclonal antibodies have proven effective in managing recalcitrant chronic rhinosinusitis with nasal polyps (CRSwNP), the concept of disease remission is evolving, underscoring the need for a comprehensive, multimodal evaluation of disease burden over time.

**Objective:**

This study first presents ultrastructural changes of the nasal epithelium in patients treated with dupilumab, assessing the potential for mucosal regeneration during long‐term follow‐up.

**Methods:**

Ten patients (*n* = 10) were enrolled. Baseline (T0) outcomes included Nasal Polyp Score, Lund‐Kennedy Score, 22‐item Sinonasal Outcome Test, Visual Analog Scale for nasal symptoms, and CT‐scan Lund‐Mackay Score. Mucosal samples were collected from the posteromedial aspect of prior antrostomies and analyzed using both light microscopy and transmission electron microscopy (TEM). Outcomes and mucosal sampling were reassessed at 12‐month (T1) and compared. Logistic bivariate analysis was performed to assess the relationship between ultrastructural tissue characteristics and clinical outcomes.

**Results:**

Significant improvements in all outcomes were observed at T1. A ciliated columnar epithelium was identifiable in most patients (*n* = 7/10). Intercellular junctions, including desmosomes, tight and adherens junctions, were evident in half of the sample (*n* = 5/10). Three patients (*n* = 3/10) showed no evidence of epithelial regrowth. Endoscopic and radiologic outcomes were significantly linked to ultrastructural findings at T1. Higher eosinophilic infiltration at T0 was a positive predictor for the presence of pseudostratified epithelium at T1.

**Conclusions:**

These first preliminary data suggest that ultrastructural response is achievable in most patients with CRSwNP. Larger cohorts are required to strengthen these findings and support the concept of disease remission in course of mAb.

## 1 Introduction

Managing patients with chronic rhinosinusitis with nasal polyps (CRSwNP) has long been a significant challenge in ENT clinical practice, primarily due to the high rate of symptom recurrence and the considerable impact on patients' quality of life (QoL) as well as on the healthcare system [1, 2]. Patients with a high type 2 inflammatory load and related comorbidities (e.g., asthma, NSAID‐exacerbated respiratory disease) are at increased risk of relapse after endoscopic sinus surgery (ESS), often leading to repeated corticosteroid use and, in some cases, steroid dependency, which may carry serious systemic consequences [3, 4]. Mucosal inflammation in CRSwNP is mainly driven by an epithelial barrier dysfunction promoting, by means of epithelial derived cytokines such as Thymic Stromal LymphoPoietin (TSLP), IL‐33 and IL‐25, a dysregulated type 2 immune response, where cytokines such as IL‐4, IL‐5, IL‐9, and IL‐13 induce inflammatory cell infiltration (including eosinophils and mast cells) and the secretion of matrix‐degrading agents (e.g., basic major protein and metalloproteinases) [5]. The identification of the disease's molecular drivers has enabled the development of targeted therapeutic strategies such as biological therapies. Dupilumab, a humanized anti‐IL‐4 receptor alpha (IL‐4Rα) monoclonal antibody (mAb), simultaneously inhibits IL‐4 and IL‐13 signaling and has demonstrated significant improvements in patient‐reported outcomes (PROs), endoscopic parameters, and sinus radiologic opacification in two phase III clinical trials and in real‐world settings [6, 7, 8]. Beyond clinical outcomes, recent analyses confirmed the effectiveness of dupilumab in reducing multiple type 2 biomarkers in nasal secretions and polyp tissue, demonstrating that IL‐4Rα antagonism suppresses key inflammatory processes, including mucosal IgE secretion and chemokine expression (e.g., exotoxin‐3) [9]. In similar settings, other authors observed a significant reduction in different inflammatory biomarkers such as fractional exhaled nitric oxide (FeNO) and nasal nitric oxide (nNO) [10], serum total IgE and urinary cysteinyl leukotrienes [11, 12]. The evidence of sustained and significant improvements in objective findings, PROs, and inflammatory biomarkers has led to the emergence of a novel concept of disease remission in the context of tailored therapy. A recent consensus by Hellings et al. proposed remission as a prolonged state of disease control, characterized by the absence of bothersome symptoms for at least 12 months, without the need for oral corticosteroids (OCS) or endoscopic sinus surgery (ESS), and without endoscopic signs of active disease [13]. However, modern personalized medicine principles advocate for a multidimensional assessment that incorporates inflammatory and histological criteria, in addition to clinical evaluation.

After an acute insult at upper airway epithelial level, physiologic tissue repair begins with an early inflammatory phase driven by IL‐6 and TNF‐α, followed by epithelial‐to‐mesenchymal transition and proliferative activation via EGF and TGF factors [14]. The final phase involves progenitor cell re‐differentiation, regulated by Notch signaling and transcription factors [15]. Chronic inflammation in CRSwNP disrupts this process, impairing both epithelial restoration and function [16]. The healthy nasal epithelium is pseudostratified with ciliated columnar cells that facilitate mucociliary clearance, supported by basal epithelial stem cells, and an underlying submucosa containing collagen, ECM, and blood vessels. Chronic inflammation leads to epithelial remodeling, loss of ciliated cells, and impaired clearance [17]. When this disease is not adequately controlled with first‐line therapies, mAbs have proven to be an effective alternative.

Although previous research has demonstrated improvements in local and circulating inflammatory biomarkers during therapy, data addressing the related histological criteria remain limited [18, 19]. MESILICO was a multicentric study that assessed the effect of anti Il‐5 on airway structural remodeling in patients treated for severe eosinophilic asthma [20]. At 12‐month evaluation, investigators observed a marked reduction from baseline in basement membrane thickness, airway smooth muscle layer thickness, epithelial damage, and tissue eosinophil counts. Similarly, the effectiveness of dupilumab in reducing airway inflammation was evaluated in a recent clinical trial (EXPEDITION; NCT02573233), which documented a decrease in inflammatory cell density within the bronchial submucosa of patients with asthma [21]. These findings suggest that, although the two agents act on different upstream mediators of the type 2 inflammatory cascade (IL‐5 and IL‐4Rα), both ultimately converge on downstream pathways that govern eosinophilic infiltration. This may explain why the resulting microscopic inflammatory patterns appear comparable across treatments. Nevertheless, no head‐to‐head studies have yet compared the ability of different mAbs to modify the histological inflammatory burden of the airway mucosa.

In this study, we aimed to provide the first preliminary evidence focused on nasal histological mucosal changes in patients treated with dupilumab. Since no prior studies have explored these aspects, this research may provide the first evidence of mucosal microscopic responses to mAb therapy for CRSwNP. Furthermore, given that the pathological basis of the disease lies within the mucosal layer, we believe that an ultrastructural assessment of the nasal epithelium may offer valuable insights into whether true disease remission, can be achieved through therapy.

## 2 Materials and Methods

This is a single‐center, prospective, interventional study conducted at Humanitas Research Hospital (Rozzano, Milan, Italy) between September 2023 and December 2024. The study was performed in accordance with the ethical standards of the Declaration of Helsinki, and it was approved by the local Ethical Committee (ref/ICH/3402). Consecutive patients treated with dupilumab for refractory CRSwNP who were willing to sign informed consent were enrolled. Clinical history and demographics were collected from medical records. Patients were referred to mAb therapy after careful evaluation by various specialists (allergists, pneumonologists, and rhinologists), and the final decision is made following a multidisciplinary board meeting. The indication to dupilumab was consistent with the requirements from the Italian regulatory agency for drugs (e.g., Agenzia Italiana del Farmaco, AIFA): (i) severe disease defined by nasal polyp score (NPS) ≥ 5 or Sinonasal Outcome Test‐22 (SNOT‐22) ≥ 50; (ii) failure or refusal of previous corticosteroid, and/or surgical treatment. The dosage of dupilumab was an initial loading dose of 600 mg, followed by 300 mg subcutaneously every 2 weeks in case of severe asthma comorbidity, or 300 mg every 2 weeks in case of severe CRSwNP only. Asthma was defined as severe when it required treatment with high‐dose inhaled corticosteroids in combination with a second controller and/or OCS [22]. All patients were using topical medications (e.g., nasal rinses and topical corticosteroids), as suggested by international guidelines [23]. All enrolled subjects underwent at least one previous ESS, embedding anterior and posterior ethmoidectomy, middle meatal antrostomies, sphenoidotomy and frontal opening (e.g., a Draf IIa procedure), preserving the physiological mucociliary drainage pattern. Those who previously underwent extended approaches, as well as mucosa‐stripping procedures (i.e., reboot surgery), were excluded. Similarly, patients with secondary CRSwNP (e.g., associated with systemic vasculitis, cystic fibrosis, or immunodeficiencies) were considered ineligible. Recruited patients were required to fill in the SNOT‐22 and Visual Analog Scales (VAS) for global sinonasal symptoms at both the baseline (T0) and 12 months from the initiation of therapy (T1). At these time points, patients underwent nasal endoscopy with assessment of NPS, Lund‐Kennedy Score (LKS), and nasal biopsy. Also, they were required to perform a CT‐scan with assessment of Lund‐Mackay Score (LMS) for sinus opacification. In accordance with previous studies, the following thresholds were considered to assess minimal clinically important differences (MCID) in patients after therapy: SNOT‐22 > 8.9; VAS‐global > 2.5; NPS > 1; LMS > 5 [24, 25]. All included subjects had suspended intranasal corticosteroids (INCS) at least 1 month prior to biopsies and in no cases were oral corticosteroids (OCS) required to control nasal symptoms throughout dupilumab therapy. Biopsies were taken under local anesthesia in the outpatient setting at the baseline (T0) and at the 12‐month examination (T1). To ensure reproducibility, all procedures followed a standardized protocol. Specimens consisted of 5 × 5 mm‐wide biopsies and were obtained from the lateral aspect of the ethmoid, close to the posteromedial wall of the maxillary antrum. Following collection, samples were fixed in both formalin and glutaraldehyde (3%), and then analyzed by two blinded reviewers using light microscopy (histochemistry, immunohistochemistry) and electron microscopy (transmission electron microscopy, TEM). In cases of disagreement, a third expert observer was consulted to reach a consensus. Sex was self‐reported by participants at the time of enrollment; gender identity was not specifically collected in this study.

### 2.1 Histochemistry

Consecutive formalin‐fixed, paraffin‐embedded tissue sections were cut at 3 μm and stained in hematoxylin/eosin for morphological assessment and eosinophil count. Picrosirius red staining was performed for 1 h at room temperature (RT) using Sirius Red (0.1% in saturated aqueous picric acid) to visualize collagen bundles. The Picrosirius Red stain was complemented with polarized light detection to analyze collagen fiber orientation, type, and spatial distribution.

### 2.2 Immunohistochemistry

Paraffin‐embedded tissue sections (3 μm) were subjected to antigen retrieval by immersion in a heat retrieval solution (DIVA Antigen Decloaker solution, Biocare Medical, USA) using a pressure cooker (Decloaking Chamber, Biocare Medical, USA). Tissue slides were treated with 3% hydrogen peroxide solution for 10 min at RT to quench endogenous peroxidase. Tissue slides were then blocked with Background Sniper (Biocare Medicals, USA) for 20 min at RT and incubated with antibodies raised against CD34 (Biocare Medical, USA, dilution 1:100), CD66b (BD Pharmingen, dilution: 1:1000), Mast Cell Tryptase (Neobiotechnologies, USA, dilution: 1:100) and Eosinophil Major Basic Protein (Biorad, Italy, dilution: 1:40) for 1 hour at RT. Tissue sections were then incubated with MACH1 HRP‐polymer for 15 min at RT (Biocare Medical USA). The chromogen reaction was developed using Betazoid DAB Chromogen Kit (BioCare Medicals) and the sections were counterstained with hematoxylin. Whole tissue slides were acquired at 20x (Objective magnification) using the automatic slide scanner Zeiss Axioscan Z1 (Zeiss, Italy).

### 2.3 Transmission Electron Microscopy (TEM)

Samples were fixed in 3% glutaraldehyde in NaCacodylate 0.1 M for 24 h at 4°C, then cut into smaller fragments, which were washed in buffer solution and post‐fixed with 2% osmium tetroxide for 1 h at RT. The samples were subsequently rinsed and stained with 2% uranyl acetate in water for 45 min. The specimens were then dehydrated and embedded in Epon‐Araldite resin, which was cured for 48 h at 60°C. All reagents were purchased from Electron Microscopy Sciences (Hatfield, PA, USA). Samples were sliced into sections of 500 nm thickness using an ultramicrotome (Leica Microsystems, Austria) and subsequently stained with toluidine blue for a complementary observation under light microscopy. For transmission electron microscopy analysis, thin sections (70 nm) were obtained using an ultramicrotome (Leica Microsystems, Austria), placed onto a copper grid, and stained with lead citrate. Observation of these sections was performed using a Talos L120 C transmission electron microscope (Thermo Fisher Scientific, MA, USA) operating at an acceleration voltage of 120 kV. The presence of the following mucosal components was assessed dichotomously and included in statistical analysis: (i) pseudostratified epithelium, (ii) cilia/microvilli, (iii) junctional complexes, (iv) apoptotic cells.

### 2.4 Statistics

Statistical analysis was conducted with SPSS (Version 28 for Macintosh, IBM). Continuous variables were presented as mean, range, and standard deviation, while discrete variables were expressed as absolute values and percentages. Non‐parametric tests were used to analyze paired data. The McNemar test was used to assess differences between dichotomous variables, while the Wilcoxon signed‐rank test was used to compare the median values of paired continuous variables. Bivariate logistic regression analysis was conducted to assess the relationship between ultrastructural microscopic characteristics and other outcomes at both time points. Odds ratios (ORs) were calculated to determine the strength of these associations. The threshold for significance was set for a *p*‐value < 0.05.

## 3 Results

### 3.1 Overview

Ten consecutive patients were enrolled in the study (M: 7/10, 70%; mean age: 57.20 ± 9.06 years, range: 35–66). Six (*n* = 6/10) of them were sensitized to at least one inhalant allergen. Six (*n* = 6/10) subjects had bronchial asthma. Between them, four patients (*n* = 4/6) presented severe asthma, and three (*n* = 3/6) had comorbid aspirin hypersensitivity (i.e., Samter's triad). None of them were smokers. The average number of previous surgeries was 3.70 ± 4.27 (range: 1–15). Mean baseline IgE and blood eosinophils were 362.37 ± 555.43 IU/mL (range: 21.3–1789.0) and 344.44 ± 194.37 cells/μL (range: 100–600), respectively. Tissue eosinophil count on nasal polyp biopsies were on average 31.00 ± 18.53 (range: 10–60) per high‐power‐field (HPF). Objective findings and PROs prior to dupilumab administration (T0) were as follows: NPS = 6.38 ± 1.59, LKS = 9.44 ± 2.79, LMS = 20.22 ± 1.92, SNOT‐22 = 56.63 ± 22.34, VAS‐global = 7.8 ± 1.76. Baseline demographic and clinical characteristics are presented in Table 1. No patients have required further surgery or needed additional OCS administration during the observation period of dupilumab treatment. Mean endoscopic and radiologic outcomes, as well as PROs, significantly improved at T1 compared to T0 (*p*‐value: NPS = 0.046; LKS = 0.035; LMS = 0.021; VAS‐global = 0.023; SNOT‐22 = 0.023). At 12‐month examination, a significant decrease of mean eosinophilic infiltration on hematoxylin/eosin staining was observed compared to baseline (mean Eos/HPF: *T*0 = 31.00 ± 18.53, *T*1 = 12.00 ± 9.1; *p*‐value = 0.014). There was an increase in blood circulating eosinophils (mean Eos/μL: *T*0 = 525.00 ± 517.55, *T*1 = 525.00 ± 517.55; *p*‐value = 0.038). Notably, no clinically relevant adverse events were reported during treatment. Table 2 summarizes clinical outcomes before and after 12 months of dupilumab treatment.

**TABLE 1:** Baseline characteristics.

Gender	Females (%)	3 (30.0)
	Males (%)	7 (70.0)
Age	Mean (years) ± SD	57.20 ± 9.06
Smoking	Yes (%)	0 (0)
	No (%)	10 (100.0)
Comorbidities	Allergy (%)	6 (60.0)
	Asthma (%)	6 (60.0)
	Severe asthma (%)	4 (40.0)
	N‐ERD (%)	3 (30.0)
Previous (1‐year) OCS use (≥ 2 cycles)	Yes (%)	2 (20.0)
	No (%)	8 (80.0)
Previous functional sinus surgeries	Mean ± SD	3.70 ± 4.27
NPS	Mean ± SD	6.20 ± 1.61
LKS	Mean ± SD	9.40 ± 2.63
LMS	Mean ± SD	20.20 ± 1.81
VAS‐global	Mean ± SD	6.65 ± 2.35
SNOT‐22	Mean ± SD	51.70 ± 22.15

Abbreviations: N‐ERD, NSAID‐exacerbated respiratory disease; OCS, oral corticosteroids.

**TABLE 2:** Outcomes statistics comparison in the overall sample.

Outcome	T0 ± SD	T1 ± SD	Effect size	*p*‐value
NPS	6.20 ± 1.61	2.50 ± 2.37	−1.84	0.046
LKS	9.40 ± 2.63	3.80 ± 2.44	−1.95	0.035
LMS	20.20 ± 1.81	7.00 ± 2.58	−2.04	0.021
VAS‐global	6.65 ± 2.35	2.78 ± 1.65	−2.02	0.023
SNOT‐22	51.70 ± 22.15	20.50 ± 7.85	−2.02	0.023
Eos/HPF	31.00 ± 18.53	12.00 ± 9.19	−2.45	0.014
Eos/μL	344.44 ± 194.37	525.00 ± 517.55	1.96	0.038

*Note:* Wilcoxon‐signed rank test was used. Effect sizes and *p*‐values are displayed.

Abbreviations: LKS, Lund‐Kennedy Score; LMS, Lund‐Mackay Score; NPS, Nasal Polyp Score; SD, standard deviation; SNOT‐22, Sinonasal‐Outcome Test‐22; VAS, Visual‐Analog Scale.

### 3.2 Transmission Electron Microscopy

**FIGURE 1:**
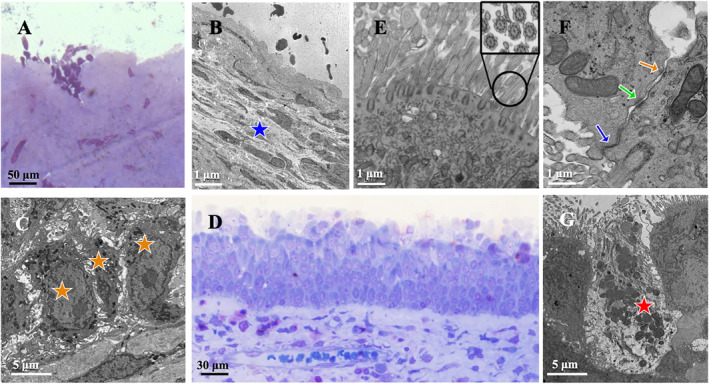
Nasal epithelium at baseline and ultrastructural changes induced by therapy. (A) Toluidine blue‐stained mucosa at T0 showing degenerated epithelium with dispersed aggregates of residual cuboidal cells and direct exposure of the subepithelial connective tissue. (B, C) Transmission electron microscopy confirms mucosal disruption with direct exposure of subepithelial layers. The basal lamina (B; blue star) is thickened, while residual epithelial cells (C; yellow star) appear degenerated, exhibiting a cuboidal arrangement, distorted cytoplasm, pycnotic nuclei, and condensed nucleoli. At T1, epithelial surface regeneration was observed in most patients (*n* = 7/10), displaying a typical pseudostratified architecture (D). In these patients, the apical domain of epithelial cells exhibited a renewed ciliary apparatus with a physiological axoneme (9 + 2 microtubule arrangement) originating from the basal layer (E; black circle highlights a transverse section). Intercellular spaces were sealed by intercellular junctions (F; blue arrow: tight junction, green arrow: adherens junction, yellow arrow: desmosome). Apoptotic cells with vacuolized, pycnotic cytoplasm were still visible in five (*n* = 5/10) patients (G; red star).

At T0, toluidine‐blue stained samples revealed a severely unstructured mucosa, degenerated into a flattened or squamous stratified epithelium lacking differentiated respiratory cells or organized cellular layers. In some areas, the epithelium was completely absent, resulting in direct exposure of the underlying extracellular matrix (ECM) to the nasal lumen (Figure 1A). Transmission electron microscopy confirmed these findings, showing disorganized nasal mucosa with a consistent loss of residual epithelial cells across all samples (Table 3). Moreover, all patients revealed disrupted intercellular junctions resulting in widened intercellular spaces (Figure 1B; Table 3). Cellular contents appeared distorted, with heterochromatic nuclei, finger‐like projections on the cellular membrane and no cilia (Figure 1C). By T1, a restored pseudostratified epithelial surface was observed in most patients (*n* = 7/10, 70%). In this group, no areas of direct exposure of the subepithelial ECM were noticed, confirming the regeneration of superficial layers in course of therapy (Figure 1D). Furthermore, with TEM, we identified the presence of a ciliary apparatus emerging from the microvilli‐rich apical surface of these cells. Structurally, cilia were consistent with their physiological arrangement characterized by a 9 + 2 coupling of microtubule doublets originating from the basal body (Figure 1E). Similarly, the lateral domains of epithelial cells exhibited a restored intercellular junction apparatus, including: (i) tight junctions, where the plasma membranes of adjoining cells fuse in a zipper‐like manner, ensuring a tight seal; (ii) adherens junctions, serving as anchoring structures that facilitate lateral adhesion between epithelial cells; (iii) desmosomes, consisting of intracellular plaques that anchor intermediate filaments and feature transmembrane glycoproteins binding to identical molecules in neighboring cells (Figure 1F). Specifically, tight and adherens were found in 7/10 (70%) patients, whereas fewer (*n* = 5/10, 50%) displayed desmosomes. Out of the whole sample, 3 (*n* = 3/10, 30%) patients still presented lack of mucosal regeneration, with compromised epithelial surface and exposure of the underlying ECM. Also, apoptotic cells were still observed in 5 patients (T0: *n* = 6/10, T1: *n* = 5/10; *p* = 0.980; Figure 1G). In 3 cases (*n* = 3/10, 30%), there was a loss of typical microvillar apicobasal polarity, possibly representing persistent ongoing inflammation‐induced epithelial dysfunction. Microscopic ultrastructural findings are displayed in Table 3 and Figure 1. Comparative analysis of most ultrastructural variables (i.e., presence of pseudostratified epithelium, cilia/microvilli and junctional complexes) between the two time points was not feasible, as they were mostly constant at baseline.

**TABLE 3:** Ultrastructural microscopic findings at both timepoints.

Patient	T0				T1			
	Pseudostratified epithelium	Cilia and microvilli	Junctional complex	Apoptotic cells	Pseudostratified epithelium	Cilia and microvilli	Junctional complex	Apoptotic cells
#1	No	No	No	No	Yes	Yes	Yes	No
#2	No	No	No	Yes	Yes	Yes	Yes	No
#3	No	No	No	No	Yes	Yes	Yes	No
#4	No	No	No	Yes	Yes	Yes	Yes^a^	Yes
#5	No	No	No	No	No	No	No	No
#6	No	No	No	Yes	Yes	Yes	Yes^a^	Yes
#7	No	No	No	Yes	Yes	Yes^b^	No	Yes
#8	No	No	No	Yes	Yes	Yes^b^	No	Yes
#9	No	No	No	Yes	No	No	No	Yes
#10	No	No	No	No	No	No	No	No

^a^ Loss of apico‐basal polarity.

^b^ Only Tight junctions and adherens.

### 3.3 Regression Analysis

At baseline, no predictivity based on endoscopic (NPS, LKS), radiologic (LMS) or patient‐reported outcomes (VAS‐global, SNOT‐22) was noticed for any ultrastructural finding. Similarly, there was no significant association between demographic characteristics and ultrastructural findings. Conversely, baseline tissue eosinophilic infiltration emerged as a positive predictor for the presence of epithelium (*p*‐value = 0.003, OR: 1.20) and cilia/microvilli (*p*‐value = 0.013, OR: 1.19) at 12‐month.

At T1, bivariate logistic analysis revealed a significant relationship between endoscopic outcomes and certain ultrastructural findings. Specifically, lower NPS values were associated with the presence of epithelium (*p* < 0.001, OR = 0.80), cilia and microvilli (*p*‐value < 0.001, OR = 0.78) and junctional complexes (*p*‐value = 0.037, OR = 0.86). Similarly, LKS was inversely associated to the same ultrastructural variables (pseudostratified epithelium: *p*‐value < 0.001, OR = 0.80; cilia and microvilli: *p*‐value < 0.001, OR = 0.80; junctional complexes: *p* = 0.022, OR = 0.84). Additionally, worse radiologic outcomes (LKS) were related to a lack of epithelial and cilia/microvilli regrowth (*p*‐value = 0.005, OR = 0.82). Notably, no associations were observed between PROs (SNOT‐22, VAS) and ultrastructural findings (*p*‐value > 0.05).

### 3.4 Immunohistochemistry

**FIGURE 2:**
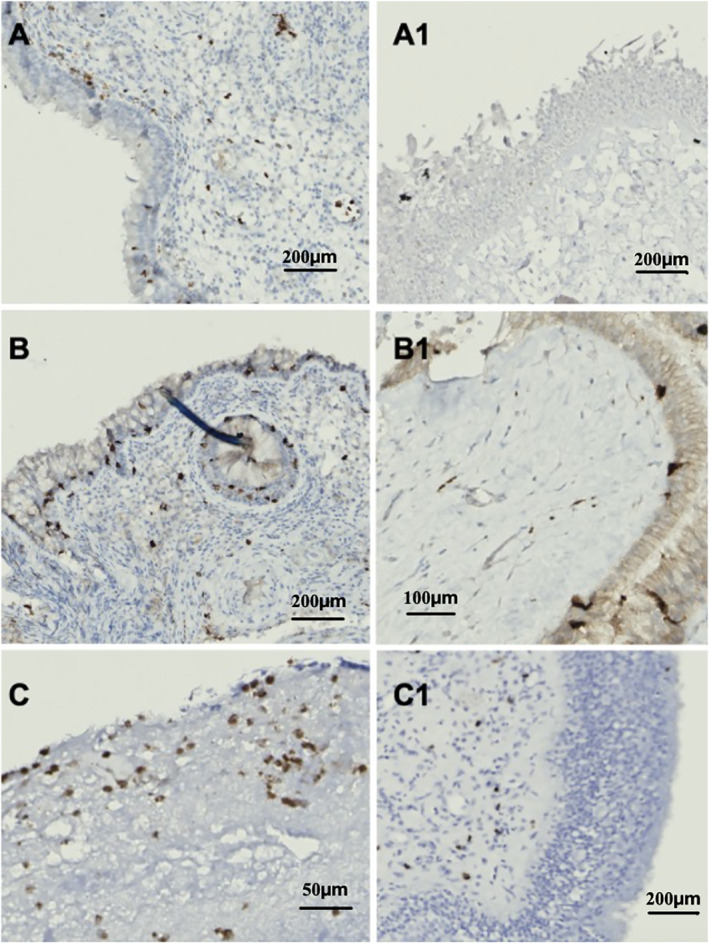
Inflammatory cells infiltrations in patients with epithelial regeneration in course of dupilumab. Neutrophil infiltration in the subepithelial connective layer, prominent at T0 (A), showed a marked reduction 12 months after treatment initiation (A1). Mast cells, which were abundant in the lamina basalis of inflamed mucosa at T0 (B), were nearly undetectable at T1 (B1). Eosinophil infiltration, significant at T0 (C), was notably diminished at T1, remaining only slightly detectable in the subepithelial layers (C1).

**FIGURE 3:**
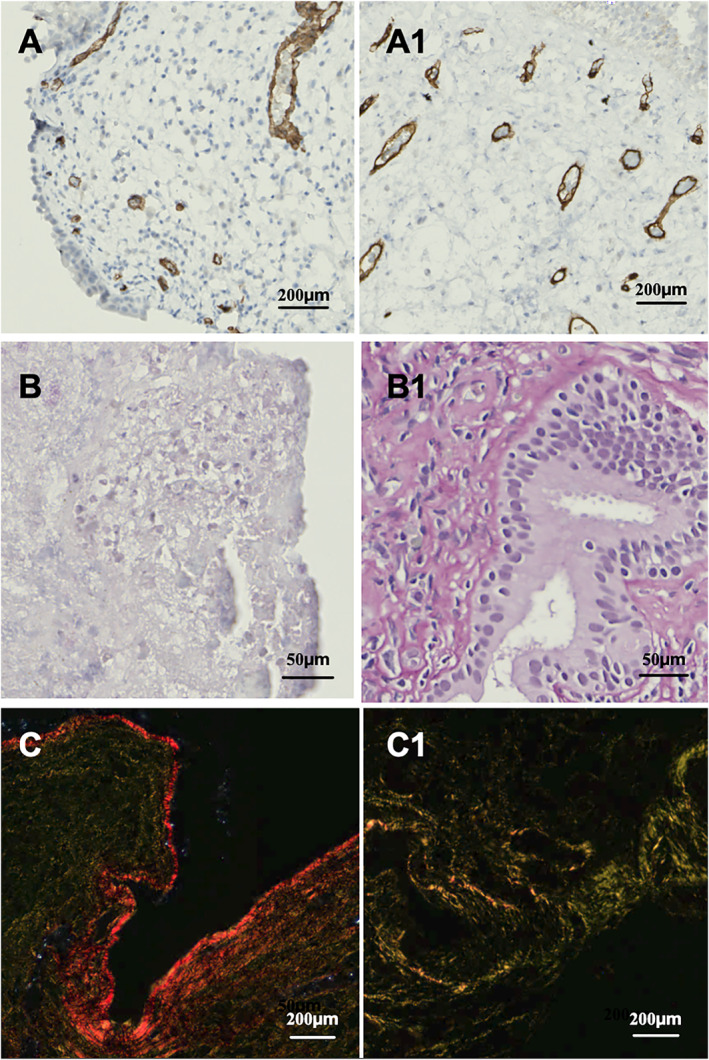
Changes in extracellular matrix components over timepoints in patients with epithelial regeneration in course of dupilumab. (A, A1) Vessels were identified using CD34 staining. At baseline (A), vascular structures appeared sparse, with the few vessels exhibiting a dilated, dysfunctional, and heterogeneous architecture. By 12 months (A1), a notable increase in vascular density was observed, along with improved homogeneity and structural organization. (B, B1) Sirius Red staining revealed an absence of detectable extracellular collagen fibrils at T0 (B). By T1 (B1), the subepithelial layers displayed significant enrichment of collagen fibrils, indicating extracellular matrix restoration. (C, C1) Polarized light microscopy highlighted a transition from predominantly “red‐type” collagen (type I) at T0 to “green‐type” collagen (type III) at T1, suggesting active subepithelial extracellular matrix regeneration.

**FIGURE 4:**
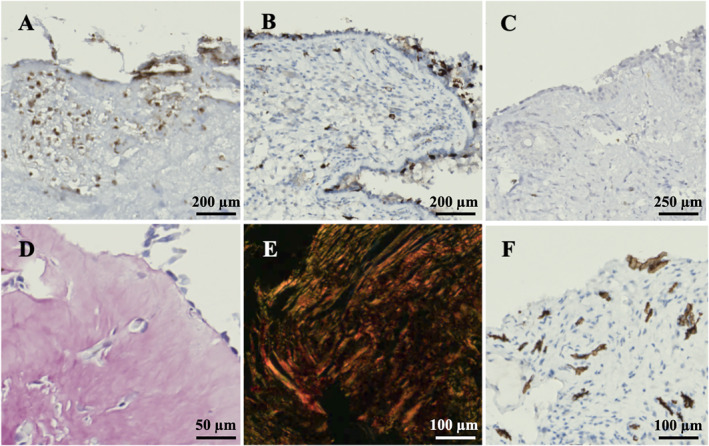
Immunohistochemical changes at T1 in patients with uncomplete epithelial regeneration at T1. Eosinophilic and mast cell infiltrate was still prominent in subepithelial layers (A, B), although neutrophils appeared reduced (C). Collagen fibrils were evident on Sirius Red staining (D), although they were predominantly red (type I) on polarized light (E). Vascular scaffold remained disarrayed (F).

At T0, inflammatory cell infiltration was prominent. Specifically, neutrophils, eosinophils, and mast cells were evenly distributed across the lamina basalis and the subepithelial layer (Figure 2A–C). By T1, a marked reduction in the infiltration of all inflammatory cell types was prominent across patients with epithelial regeneration (Figure 2A1–C1). In those samples, noticeable improvements were observed in various components of the ECM. On CD34 staining, the vasculature, which appeared enlarged and disorganized at T0, showed a markedly improved architecture and greater homogeneity by T1 (Figure 3A,A1). Collagen fibrils, which were sparse in T0 specimens, had been replenished, restoring the subepithelial extracellular matrix at T1 (Figure 3B,B1). Additionally, polarized light microscopy demonstrated a transition from red (type I) to green (type III) collagen fibrils (Figure 3C,C1). Patients whose epithelial regeneration was absent at T1 (#5, #9, #10; *n* = 3/10, 30%) still presented with a markedly high number of eosinophils and mast cells on the subepithelial layer at T1, whereas neutrophils were slightly reduced (Figure 4A–C). Collagen fibrils had a higher density compared to T0, although they remained predominantly red (type I) on polarized light (Figure 4D,E). Similarly, in this group, microvascular structures appeared disarrayed and inhomogeneous (Figure 4F).

## 4 Discussion

Our study aimed to assess microscopic ultrastructural changes in the sinonasal mucosa of patients undergoing treatment with dupilumab and to explore correlations between PROs, endoscopic parameters, and ultrastructural characteristics. At baseline, ultrastructural findings were homogeneous across the entire sample, showing unstructured mucosa with scarce residual epithelial cells and disrupted intercellular junctions. After 12 months of dupilumab treatment, most of our patients exhibited pseudostratified ciliated epithelial regrowth, confirming the improvements we observed for objective and patient‐reported parameters. Notably, every assessed variable met the MCID, with significant effect sizes. As expected, hematoxylin/eosin staining revealed a significant reduction in tissue eosinophils/HPF compared to baseline. This aligns with the literature, as the inflammatory cascade mediated by IL‐4 and IL‐13 contributes to the synthesis of chemokines and adhesion molecules, regulating inflammatory cell migration. Specifically, Il‐4Rα blockage results in reduced vascular cell adhesion molecule‐1 (VCAM‐1) expression, eosinophil adhesion to the vascular endothelium, and subsequent tissue migration [26]. While these cytokines do not directly regulate eosinophil maturation, reduced tissue migration may account for the transient peripheral blood eosinophilia observed during treatment, which remained clinically insignificant in our cohort, in line with most series reported in the literature [27]. Nasal mucosa regeneration was recently demonstrated by Pirola et al. in patients with severe CRSwNP who underwent extended mucosa‐stripping approach (e.g., reboot surgery), revealing the central role of the epithelium in the pathogenic mechanisms of disease [28]. To ensure reproducibility and maintain methodological continuity with previous findings, specimens were collected in an analogous fashion, specifically sampling the posteromedial aspect of prior antrostomies. Our results align with their findings, although derived from a population treated with a non‐surgical approach, nonetheless targeting the underlying inflammatory mucosal substrate. Immunohistochemistry was consistent with ultrastructural findings: Sirius Red staining revealed increased collagen fibril deposition in the ECM at T1, while polarized light confocal microscopy demonstrated a higher concentration of type III collagen, forming an elastic network supporting ECM neo‐synthesis. In contrast, type I collagen, a stiffer fibrillar protein, likely reflects a lower ECM turnover rate [29]. Junctional complexes, defined by the coexistence of desmosomes, tight and adherens junctions, were correctly expressed in half of the sample. Additionally, two more patients exhibited tight and adherens junctions but lacked desmosomes. The reassembly of intercellular junctions within regenerating epithelium remains poorly understood, likely requiring functional epithelial cells, active protein synthesis, and an intact exocytotic apparatus. In the intestinal system, restoration of junctional complexes has been linked to ion channel function (e.g., ClC‐2), which may regulate intercellular connections [30]. The lack of desmosomes we observed in patients with regrown pseudostratified ciliated epithelium may indicate residual dysfunction affecting complex intracellular pathways. Overall, after dupilumab treatment, 30% of patients still exhibited disorganized, unstructured mucosa lacking epithelial regeneration and direct exposure of the ECM to the nasal lumen. This may be attributed to persistent mucosal edema or polypoid degeneration in the sampled region, along with reduced epithelial regeneration capacity. Interestingly, histopathologic specimens of patients with no epithelial regrowth after 12 months of treatment were sampled from patients with lower eosinophils/HPF count at baseline. Accordingly, regression analysis confirmed that tissue eosinophil count at baseline was a significant predictor of epithelial and ciliary regeneration after treatment. Based on these findings, we assume that the higher is type 2 inflammation, the higher is the expression of tissue eosinophils, with an increased likelihood to benefit from dupilumab treatment, in line with recent evidence emerging form molecular endotyping of nasal brushing [31]. Nonetheless, effective predictive histological biomarkers remain elusive, and our findings cannot yet provide conclusive evidence due to the small sample size and the preliminary nature of the data, necessitating larger cohorts for confirmation. Our findings align with a recent analysis by De Corso et al., which observed that local eosinophil count was associated with greater symptom severity, whereas blood eosinophilia showed no correlation with higher QoL burden [32]. Similarly, research by Dorismond et al. demonstrated that elevated tissue eosinophils significantly predicted increased dupilumab prescriptions in CRSwNP, although their study did not specifically focus on treatment response prediction [33]. Worse endoscopic and radiologic outcomes (NPS, LMS) were negative predictors for ultrastructural improvements after 12 months of treatment. However, no clear association was observed between these findings and PROs. This aligns with existing literature. A recent meta‐analysis by Jeong et al. found that commonly used nasal polyps endoscopic scoring systems (e.g., NPS, LKS, Lilholt score, Meltzer score) do not correlate with PROs such as SNOT‐22, nasal congestion scores, or objective measures of olfaction [34]. Similarly, real‐world evidence supports the correlation between endoscopic and radiologic findings, although this association is lacking for subjective outcomes [35]. This may reflect the complex interplay between anatomical improvements and symptom perception, which is likely influenced by individual patient sensitivity.

This study has several limitations. First, it was designed as a pilot study; accordingly, the small sample size and the absence of a control group receiving alternative treatment (e.g., surgery) limit the generalizability of our results. Secondly, as recent literature data have highlighted the possibility of clinical remission during prolonged treatment, further studies with an extended follow‐up will be required not only to confirm but also to further delineate the ultrastructural modifications associated with prolonged treatment [36]. It is essential to recognize that the high direct and indirect costs associated with electron microscopy and immunohistochemistry limit the feasibility of large‐scale studies and their routine clinical implementation. Importantly, we acknowledge as a further limitation that epithelial components were scored dichotomously, which may have introduced a degree of bias. This approach was adopted to enhance statistical feasibility and enable logistic regression analyses. Moreover, it should be noted that, to date, no definitive criteria have been established to assess ultrastructural responses in patients treated with mAbs. From this perspective, future studies are warranted to validate reliable and reproducible quantitative tools for evaluating mucosal responses during treatment. Similarly, the assessment of immunohistochemical findings was primarily qualitative; however, its consistency with ultrastructural analyses, reviewed independently and blindly, supports its validity. Nevertheless, the use of TEM allowed us to preliminarily reveal the hidden aspects of mucosal response to dupilumab, confirming that improvements in patient‐reported, endoscopic and radiologic outcomes may be supported in most patients by the regrowth of a physiologic ciliated epithelium. Future perspectives include designing comparative analyses to evaluate changes in the inflammatory infiltrate across different epithelial samples, including non‐respiratory mucosa (e.g., olfactory epithelium), as well as among patients treated with diverse mAbs. Furthermore, additional studies are required to provide evidence of ultrastructural modifications in patients with secondary CRSwNP, particularly in those exhibiting specific, defined histopathologic patterns such as eosinophilic granulomatosis with polyangiitis (EGPA). The strength of this study lies in providing the first ultrastructural evidence of nasal epithelium regeneration during dupilumab therapy, thereby supporting a multidimensional concept of disease remission. These findings should be interpreted within the context of limited clinical experience, highlighting the need for larger, multicenter studies to confirm and expand upon these results.

## 5 Conclusions

This study provides the first ultrastructural evidence of nasal epithelium response to dupilumab. Investigating mucosal regeneration beyond the resolution limits of light microscopy enhances our understanding of tissue repair and may hold implications for other anatomical sites. Although based on preliminary data, our findings support the efficacy of dupilumab in promoting epithelial restoration, despite the persistence of structural abnormalities in some patients. All patients showed clinical improvement, despite persistent ultrastructural abnormalities in some cases. This mismatch between tissue findings and PROMs suggests that symptom burden may be often influenced by factors beyond mucosal structure. Greater mucosal regeneration was observed in patients with a stronger type 2 inflammatory profile, highlighting the importance of patient selection. Larger studies with longer follow‐up are needed to confirm these results and better to understand the mechanisms of epithelial repair in CRSwNP.

## Author Contributions

**Francesco Giombi:** writing – original draft, investigation, methodology. **Gian Marco Pace:** writing – original draft, conceptualization. **Elena Vezzoli:** formal analysis, visualization. **Fabio Grizzi:** data curation. **Fabio Pasqualini:** data curation. **Michele Cerasuolo:** resources. **Enrico Heffler:** writing – review and editing. **Giovanni Paoletti:** writing – review and editing. **Francesca Puggioni:** writing – review and editing. **Giuseppe Mercante:** validation. **Giuseppe Spriano:** supervision. **Giorgio Walter Canonica:** supervision, **Luca Malvezzi:** project administration, funding acquisition.

## Funding

The publication fee for this work was funded by IRCCS Humanitas Research Hospital.

## Conflicts of Interest

Enrico Heffler reports fees for speaker activities and/or advisory boards participation from Sanofi, Regeneron, GSK, Novartis, AstraZeneca, Chiesi, Almirall, Bosch Healthcare, Lofarma, Orion Pharma, Celltrion‐Healthcare, Apogee Therapeutics, Blueprint Medicines, Gentili, Firma outside the sumitted work. Giovanni Paoletti reports fees for speaker activities and/or advisory boards participation from Lofarma, GSK, and AstraZeneca, outside the submitted work. Giorgio Walter Canonica reports research or clinical trials grants paid to his Institution from Menarini, AstraZeneca,GSK, Sanofi Genzyme and fees for lectures or advisory board participation from Menarini, AstraZeneca, CellTrion, Chiesi, Faes Farma, Firma, Genentech, Guidotti‐Malesci, GSK, HAL Allergy, Innovacaremd, Novartis, OM‐Pharma, Red Maple, Sanofi‐Aventis, Sanofi‐Genzyme, Stallergenes‐Greer and Uriach Pharma, outside the submitted work. The other authors declare no conflicts of interest.

## Data Availability

The data that support the findings of this study are available on request from the corresponding author. The data are not publicly available due to privacy or ethical restrictions.
